# Longitudinal Monitoring of Inflammatory Bowel Disease in Mice Using Endoscopic Optical Coherence Tomography

**DOI:** 10.1093/ibd/izaf045

**Published:** 2025-03-10

**Authors:** Muktesh Mohan, Oana-Maria Thoma, Shivani Sharma, Gargi Sharma, Markus F Neurath, Maximillian Waldner, Kanwarpal Singh

**Affiliations:** Max-Planck-Institut für die Physik des Lichts, Erlangen, Germany; Max-Planck-Zentrum für Physik und Medizin, Erlangen, Germany; Max-Planck-Zentrum für Physik und Medizin, Erlangen, Germany; First Department of Medicine, Friedrich-Alexander-Universität Erlangen-Nürnberg, Universitätsklinikum Erlangen, Erlangen, Germany; Max-Planck-Institut für die Physik des Lichts, Erlangen, Germany; Department of Physics, Friedrich-Alexander-Universität Erlangen-Nürnberg, Erlangen, Germany; First Department of Medicine, Friedrich-Alexander-Universität Erlangen-Nürnberg, Universitätsklinikum Erlangen, Erlangen, Germany; First Department of Medicine, Friedrich-Alexander-Universität Erlangen-Nürnberg, Universitätsklinikum Erlangen, Erlangen, Germany; Max-Planck-Zentrum für Physik und Medizin, Erlangen, Germany; First Department of Medicine, Friedrich-Alexander-Universität Erlangen-Nürnberg, Universitätsklinikum Erlangen, Erlangen, Germany; Max-Planck-Institut für die Physik des Lichts, Erlangen, Germany; Max-Planck-Zentrum für Physik und Medizin, Erlangen, Germany; Department of Physics, Friedrich-Alexander-Universität Erlangen-Nürnberg, Erlangen, Germany; Department of Electrical and Computer Engineering, McMaster University, 1Hamilton, ON, Canada

**Keywords:** endoscopic optical coherence tomography, inflammatory bowel diseases (IBD), colitis, dextran sulfate sodium (DSS), attenuation coefficient, image segmentation, endoscopic probe for OCT

## Abstract

**Background:**

Inflammatory bowel disease (IBD) is one of the fastest-growing diseases globally. Nearly 5 million people are affected by IBD, with an incremental growth rate of 47.45% between 1990 and 2019.

**Aim and Methods:**

We aim to provide a noninvasive approach to detecting IBD with an in-house developed 1310 nm endoscopic optical coherence tomography (OCT) system. Mice with acute colitis underwent a longitudinal colon imaging process for real-time and long-run disease progression. The OCT images were processed and segmented using a computer vision image processing-based segmentation algorithm for further thickness mapping and attenuation coefficient calculations.

**Result:**

An increase in overall colon wall thickness due to inflammation was observed, as well as a reduction in attenuation coefficient due to a change in refractive index.

**Conclusion:**

Comparable results with white light endoscope and histological examination suggest the clinical potential of the 1310 nm endoscopic OCT system for in vivo assessment of IBD.

## Graphical Abstract

**Graphical Abstract:**
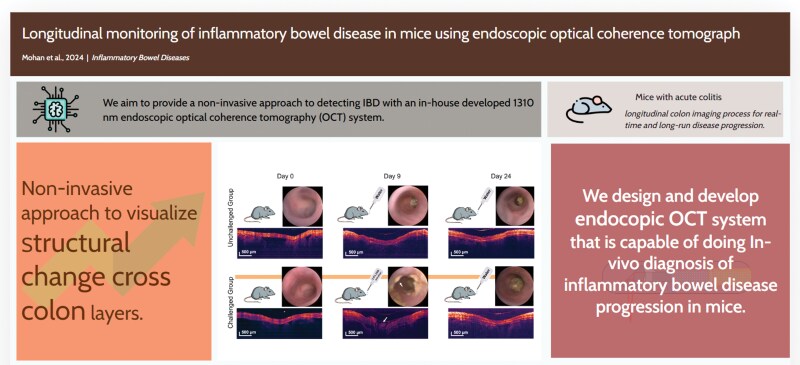

**Key Messages**

**What is already known?**

Inflammatory bowel disease (IBD) is generally diagnosed using white light endoscopy, and the information in colon wall microstructure was observed using a histopathology test.

**What is new here?**

The endoscopic optical coherence tomography provides cross-sectional and microstructural information about the colon wall, which the white light endoscope can’t achieve. Also, the current study can quantify the level of inflammation using a statistical approach noninvasively.

**How can this study help patient care?**

This study significantly enhances our understanding of IBD, providing a new tool for physicians to deliver more accurate treatment. Ultimately, this noninvasive approach can lead to more targeted and effective patient therapies, offering hope for improved outcomes.

## Introduction

Inflammatory bowel disease (IBD) is an umbrella term used to describe chronic inflammation of digestive tract tissue.^1^ IBD is characterized by abdominal pain, rectal bleeding, diarrhea, weight loss, and fatigue.^2^ For some people, IBD presents itself with mild symptoms, but for others, its debilitating condition leads to life-threatening complications. To diagnose IBD, a person usually undergoes a combination of test procedures that includes lab testing (blood and stool test) and endoscopic procedures (colonoscopy, upper endoscopy, flexible sigmoidoscopy) or imaging procedures (X-ray, CT-scan, Magnetic Resonance Imaging (MRI)).^3^ One of the most frequently used tools to diagnose IBD is the white light endoscopy, which unfortunately provides only surface information. However, it is well known that IBD affects not only the superficial layers of the intestine but also the deeper structures.^4^ To overcome this limitation of white light endoscopy, an endoscopic swept-source optical coherence tomography (SS-OCT) system able to perform cross-sectional colonoscopic imaging in the mouse intestines is presented, which is critically important for prognostics and treatment.

Optical coherence tomography (OCT) is based on low coherence interferometry,^5^ which provides cross-sectional and morphological information of biological samples. OCT has evolved in the past 3 decades and found applications in the various fields of biomedical imaging. There are several variants of OCT, such as time domain OCT, Fourier domain OCT, swept-source OCT, and full-field OCT. Swept-source OCT outperforms other variants in terms of sensitivity and speed. Endoscopic SS-OCT is a noninvasive technique that can provide high-resolution 3-dimensional (3D) microstructural information of the colon wall in real-time and in situ environments.^6^

Other methods have been adapted previously for assessing colitis in mice models, such as contrast-enhanced μCT,^4^ multiphoton endomicroscopy,^7,8^ and optoacoustic microscopy.^9,10^ While these techniques provide valuable information in the visualization of some aspects of inflammation, such as changes in vascularity or immune cell infiltration, the assessment often relies on the evaluation of a pre-selected area of interest to determine changes in the gut. The current imaging system stands out by offering noninvasive imaging capabilities while ensuring comparative resolution that rivals existing technologies (Table 1). Additionally, it claims an impressive imaging depth, allowing for greater penetration into tissues, and achieves higher imaging speeds, which knowingly enhances the efficiency of clinical assessments.

**Table 1.** Comparison between other imaging modalities and current endoscopic SS-OCT.

	Contrast-enhanced µCT^4^	Multiphotonendomicroscopy^7,8^	Optoacoustic microscopy^9^	Current endoscopic SS-OCT
**Axial resolution**	5-30 µm	2-2.5 µm	5-10 µm	5 µm
**Lateral resolution**	10-295 µm	30-60 µm	20-40 µm	20 µm
**Imaging depth**	200 µm	200 µm	3 mm	2.8 mm
**Imaging speed and time**	Up to 30 fps 17 sec to 4.5 min	1 fps 20-40 sec/stack	5 min/stack	40 fps 2 min

In this study, we developed an endoscopic SS-OCT to assess the colitis development in mice exposed to dextran sodium sulfate (DSS). Furthermore, the mice were monitored during the recovery phase as well. We developed a segmentation technique for the colon wall segmentation during the post-processing of the data and utilized it to quantify colon inflammation. The progression of colitis was assessed based on a quantitative analysis of intestine wall thickness and the attenuation coefficient of the intestine tissue. Statistical analysis was performed on the progression of the disease from the induction of DSS for colitis to the peak of colitis and up to the recovery time.

## Materials and Methods

### Endoscopic Swept-Source OCT System

**Figure 1.**
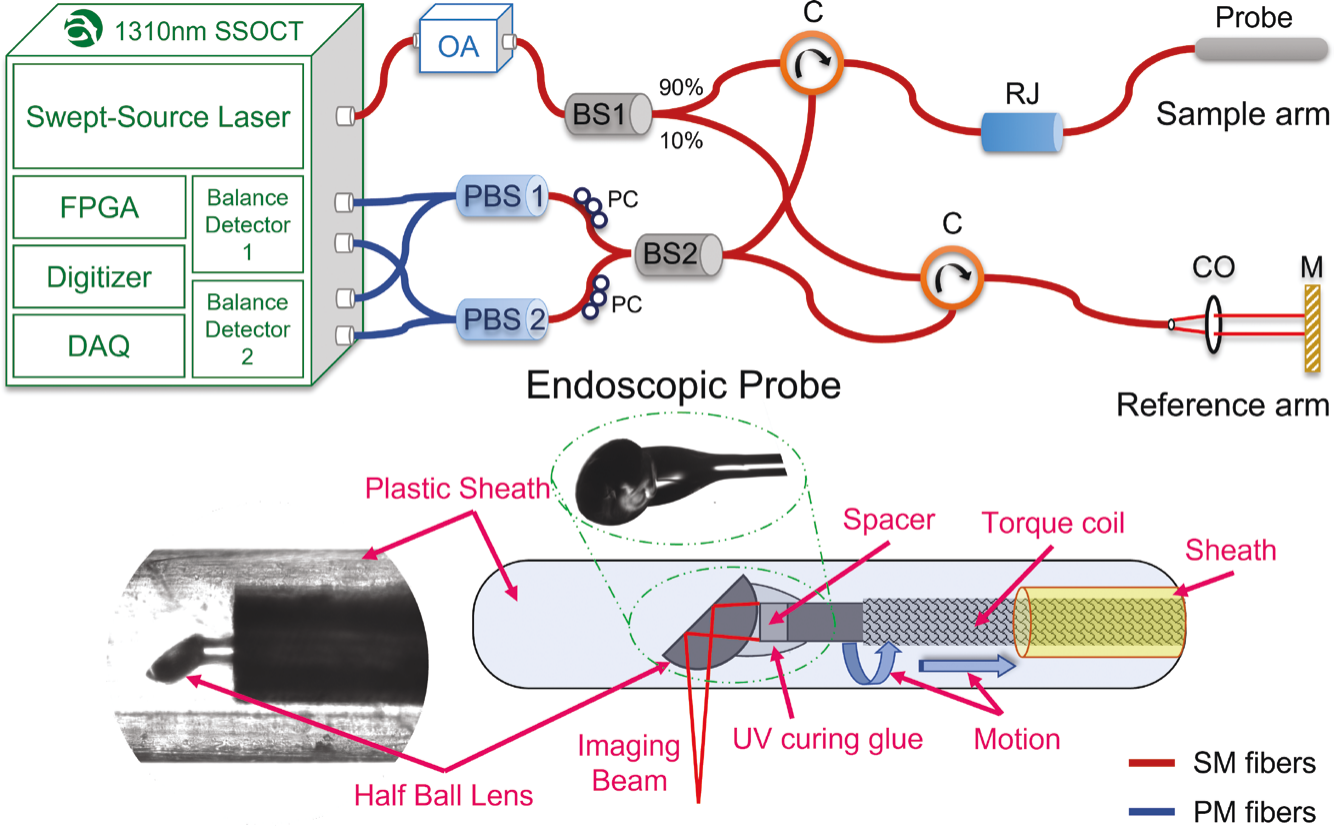
Schematic diagram of swept-source optical coherence tomography system with an endoscopic probe. OA, optical amplifier; BS, fiber beam splitter; C, circulator; CO, collimator; DAQ, data acquisition system; M, mirror; PBS, polarization beam splitter; PC, polarization controller; PM fiber, polarization maintaining fiber; RJ, rotary junction; SM fibers, single mode fiber. The Inset picture shows the microscopic image of the in-house built endoscope probe tip.

A portable endoscopic SS-OCT system shown in Figure 1 is designed in-house^11^ with off-the-shelf optics and a commercially available swept-source OCT engine provided by Axsun Technologies. OCT system has a sweep source laser with 1310 nm central wavelength, a bandwidth of 140 nm, and a wavelength sweeping rate of 100 kHz, providing 24 mW average output optical power.^12^ An optical amplifier (Series CLD1000, ModelCLD1015, Thorlabs Inc. BOA1130S, Thorlabs Inc.) compensates for the optical power losses from the optical components. Light coming from the optical amplifier splits into 2 parts, where 90% of the power goes to the sample arm through a circulator, and 10% goes to the reference arm through another circulator.^12^

The sample arm consists of 2 major components: a rotary junction (RJ) and an endoscopic probe. The reference arm consists of a collimator and a reference mirror. The distance between the mirror and the collimator is kept variable to find the optimal distance for the constructive interference between reflected and backscattered beam from the reference and the sample arm, respectively.^13^ The interference signal splits into 2 parts with a 50:50 beam splitter (BS2) for balanced detection. To minimize the polarization effects and make the detection polarization-independent, polarization beam splitters and a polarization controller are incorporated here based on our previous work.^12^ Optical fiber-based OCT systems suffer from polarization artifacts in the image.^14^ This happens because of a random change in the polarization of the light as the probe is rotating. Balance detection through 2 polarization beam splitters makes the system polarization-independent. The balanced photodetectors are connected to a data acquisition (DAQ) card, digitizer, and field programable gate array. The high-speed data processing occurs within the module, giving depth-encoded B-scan intensity image as an output. The sensitivity of the system was measured to be 103 dB.

### Endoscopic Probe and RJ

The probe is designed and developed in-house. Single mode (SMF-28) fiber is used for the probe fabrication. To make a spacer, a 250 µm diameter multimode fiber (FG105LCA, Thorlabs) with a 105 µm core is spliced with an SMF-28 fiber.^11^ A half-ball lens is attached to the tip of the spacer at an angle of ~45º with the help of ultraviolet (UV) curing optical glue. Further, fiber is placed inside the torque coil, made up of a flexible metal jacket to provide mechanical support during the rotatory motion. The fiber and torque coil are fixed with the UV curing glue to increase strength and reduce slip motion. Then, the torque coil is placed inside a flexible plastic sheath and rotates without hindrance. Another end of the fiber is connected with the FC/APC (angled physical contact) connector, and a metal tubing is also connected at the FC/APC end to strengthen the probe. The half-ball lens end of the fiber is placed inside clear plastic tubing (sheath) to isolate the sample surface from the ball lens as shown in Figure 1. The probe has an overall length of 1730 mm, making it flexible for clinical applications.

The FC/APC connector of the probe is connected to the RJ, which rotates the fiber at a synchronous speed of 2400 rpm, and a pullback translation stage is attached to the RJ. The pullback speed of 0.1 mm/sec with rotation gives a helical scanning profile of the colon or any other tube-like biological sample surface. In this study, we measure a back scanning length of 12 mm, which can be increased according to application requirements. The total time required for one set of DAQ is 120 sec.

### DSS-Induced Colitis Model

Wild-type mice with a C57BL/6J background were bred in-house and exposed to the DSS colitis model. A 2.0% DSS solution (reagent‐grade, mol. wt. 36 to 50 kDa; MP Biomedicals, Cat. No. 02160110) was prepared in drinking water. The mice exposed to the colitis model received DSS solution for 7 days, followed by 2 days of regular drinking water. The control mice only received drinking water for 9 days. To assess the colitis development, the mice were weighed daily. Furthermore, endoscopic (Karl Storz Coloview) and OCT measurements were performed before the start of the model (day 0), at the peak of the inflammation (day 9), and after the mice recovered (day 24). The mice were anesthetized using 1.5%–2.0% isoflurane for endoscopic and OCT measurements. Inflammation development was assessed using the MEICS scoring system.^15^

### Histological Analysis

To allow microscopic examination of the entire length of the colon, so-called Swiss rolls were prepared by opening the entire colon longitudinally and rolling with the mucosa outward. Samples were embedded in paraffin and stained with hematoxylin and eosin (H&E staining). A white light microscope was used to acquire the histological images of the sample slides.

### Thickness Profiling and Attenuation Coefficient Calculation of OCT-Acquired Images

The thickness of the colon wall was evaluated along the scanning length of the colon. Colon wall includes muscularis propria, submucosa, muscularis mucosa, and colonic mucosa layers. From the stack of segmented B-scan images, an orthogonal cross-sectional view was extracted in the YZ plane that gives us the thickness profile of the colon wall along the whole imaging length of 12 mm. Thickness was then measured at 10 different locations of the colon wall, and further statistical analysis was performed.

The attenuation coefficient was calculated based on the Beer-Lambert law for the dispersion of light inside a scattering media.^16^ Based on the inflammation of the tissue, scattering property may change, leading to variation in the overall attenuation of light inside the tissue/colon. For the OCT system, Beer-Lamberts law can be formulated as:


(1) $$\begin{array}{l} I=I_{0}e^{-2\left(\mu_{s}+\mu_{a}\right)z} \end{array}$$

where *I* is the intensity of light represented by the A-scan profile, *I*_0_ is the peak intensity at the sample’s surface, $\mu_{s}$ is the scattering coefficient, $\mu_{a}$ is the absorption coefficient, and *z* is the imaging depth. Multiplication of 2 is for the round-trip journey of the imaging light beam. Attenuation coefficient $\mu_{A}$ is the scattering and absorption coefficient sum.


(2) $$\begin{array}{l} \mu_{A}=\;\mu_{s}+\mu_{a} \end{array}$$

The coefficient value of the exponential curve fitting of the A-scan gives the attenuation coefficient, which was calculated over the scanning length of the colon, and statistical analysis (2-way **Analysis of variance formula **(ANOVA)) was performed.

## Results

### Colon Wall Segmentation and Post-Processing of OCT Data

Helical scanning of the mice colon results in a sequence of A-scan intensity stacks as output data from the OCT system. A post-processing is required to get a 2D B-scan image sequence. The number of A-scan per B-scan is synchronized with the rotating speed of the RJ. Based on the synchronization, B-scan stacks were extracted and further processed.

**Figure 2.**
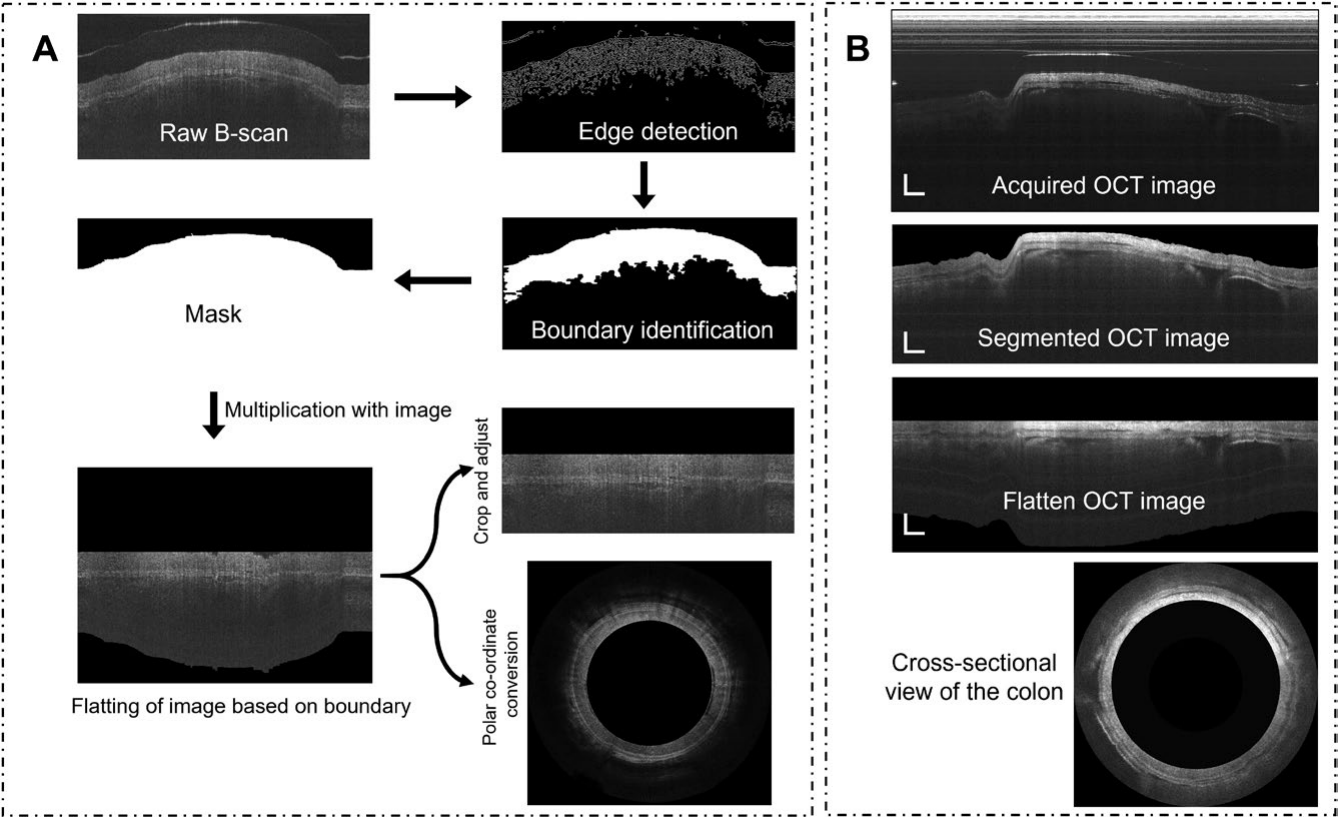
**A,** Steps involved in computer vision image processing-based boundary layer segmentation algorithm developed in MATLAB. **B,** OCT images of challenged mice’s colon during segmentation steps (Scale: 500 µm). OCT, optical coherence tomography.

B-scan images provide the cross-sectional view of the colon wall and the transparent plastic sheath. When the endoscope is inserted into the colon of mice, the contact between the colon wall and probe sheath is sometimes lost, and uneven morphology can be seen in the B-scan profile, as shown in Figure 2A (raw B-scan). A MATLAB algorithm was developed based on a computer vision image processing tool to remove the unwanted gap and get a smooth surface profile. A raw B-scan image was acquired as a first step, and then a complex computer vision edge detection algorithm, “canny,” was used to extract the boundary of the colon wall.^17^ The edge detected image is further filled with holes. Morphological operations, including dilation and erosion, were subsequently applied to enhance the clarity of the detected boundaries. This series of transformations resulted in a distinct and well-defined demarcation of the boundaries in the image.

A binary mask was created out of it based on the region of interest. The binary mask is then multiplied by the raw B-scan image and flattened based on the mask boundary. The flattened image is cropped and saved for further processing. One set of the flattened data is converted into a circular disk shape B-scan profile similar to the original colon shape. The circularly generated B-scans were compiled together to form a 3D colon image. To visualize an enface image from different depths of the colon wall, segmentation and flattening the image are necessary. It enables visualization of features beneath the top surface, such as crypts and blood vessels, and accurate quantification of colon wall thickness.

Figure 2B shows the improvement in the OCT images for more accurate quantification of different parameters after segmentation. Figure 2B represents the OCT images of the challenged group of mice. Background noises and uneven curves present in the raw B-scan images caused by helical scanning along with motion artifacts during DAQ can be suppressed. The final result of this algorithm gives much more significant dataset for further quantification and visualization.

### Endoscopic SS-OCT Imaging of Mice Colon

**Figure 3.**
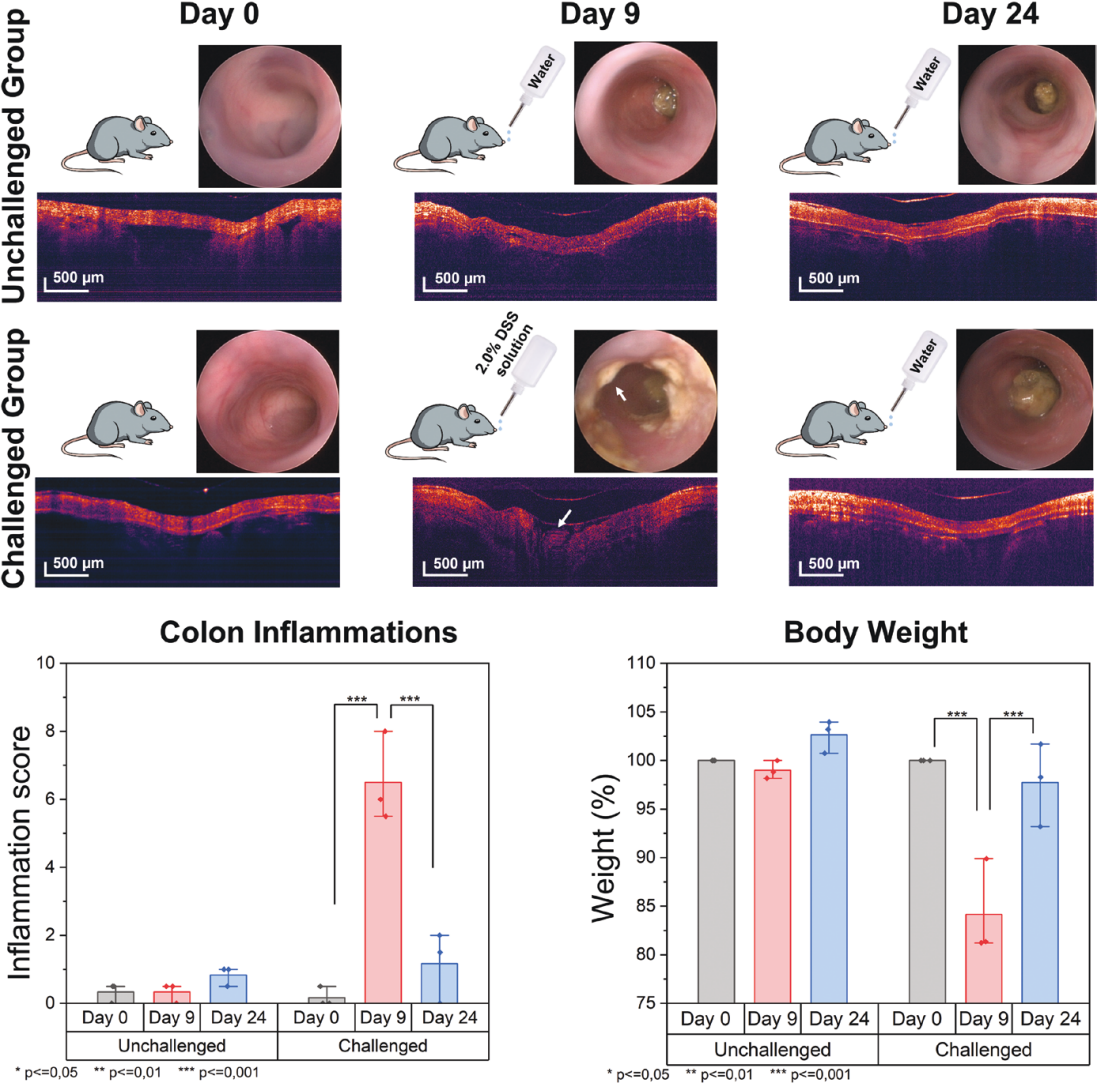
Schematic representation of the whole study along with white light and OCT endoscopic imaging at different stages of the experiment (arrow mark shows the colitis in colon); plot shows the significant colon inflammation score and body weight of challenged and unchallenged mice over the period of the experiment. OCT, optical coherence tomography.

An endoscope probe of diameter 2.3 mm was inserted into the colon of anesthetized mice, and SS-OCT images were acquired. The helical scanning was performed for a pullback distance of 12 mm. The number of A-scans per B-scan was 5012, and the total number of B-scans for a distance of 12 mm was 4050. Total 6 mice were divided into 2 groups and imaged with the white light endoscope and OCT endoscopic probe. A schematic representation of the procedure is shown in Figure 3 with the colon inflammation score and body weight count plot with respect to experimental days.

On day 0, all mice were imaged using a white light endoscope and OCT endoscope and showed the normal condition of their colon. The DSS colitis model group, also referred to as the challenged group, showed high inflammation in the colon on day 9, as described by both weight and endoscopic assessments. At the peak of colitis (day 9), the challenged mice showed high inflammation in the gut (*P* < =.001) with significant weight loss (*P* < =.001) compared to the unchallenged ones. Interestingly, OCT-acquired images also revealed differences in the appearance of the colon of challenged mice on day 9, whereas no change could be observed in an unchallenged group of mice at the same time. During the recovery phase of the challenged group, mice drank normal water instead of 2.0% DSS solution. OCT images were acquired along with a white light endoscope for up to 24 days, and changes were recorded.

**Figure 4.**
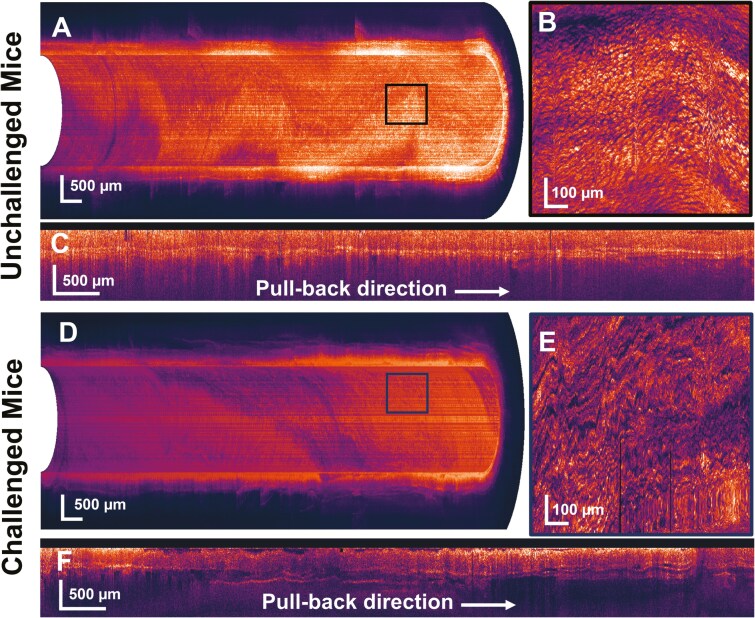
**A,** Three-dimensional OCT image of half mouse colon along its length. **B,** Enface image of the epithelial layer at marked position. **C,** Cross-sectional view of unchallenged mouse colon along the scanning length. **D,** Three-dimensional OCT image projection of challenged mice. **E,** Enface image projection of epithelial layer at marked position. **F,** Cross-sectional view of challenged mouse colon along the scanning length. OCT, optical coherence tomography.

A 3D projection of the acquired OCT data gives the perspective of visualizing the in vivo condition of the mouse colon. We can observe the real-time condition beneath the colon layers. Figure 4B shows the epithelium layer’s enface image, which shows the presence of microstructures identified as crypts. The high-resolution image with more positioning clarity is compromised due to motion artifacts present during high-speed rotation on the fiber probe, although a high degree of motion artifacts was removed during data post-processing. Compared with unchallenged mice colon, the challenged mice have more microstructure deformities, as shown in Figure 4E. Due to colitis, the crypt structures were destroyed, and the overall thickness of the colon increased by the impact of inflammation, as shown in Figure 4F.

### Correlation Between OCT and Histological Images

A comparative study was performed between OCT and histology images of the mice colon. Histology is widely used as the gold standard for diagnosis of mucosal changes in the gut. Unfortunately, histology is an invasive process where tissue has been removed from its definite position, whereas OCT is a noninvasive process that provides optical biopsies of the tissue in vivo without any excision. OCT images were compared with the corresponding hematoxylin and eosin stain images. The data from OCT images and Hematoxylin and eosin (H&E) staining were compared in 4 different layers of the murine colon: muscularis propria, submucosa, muscularis mucosa, and colonic mucosa.

**Figure 5.**
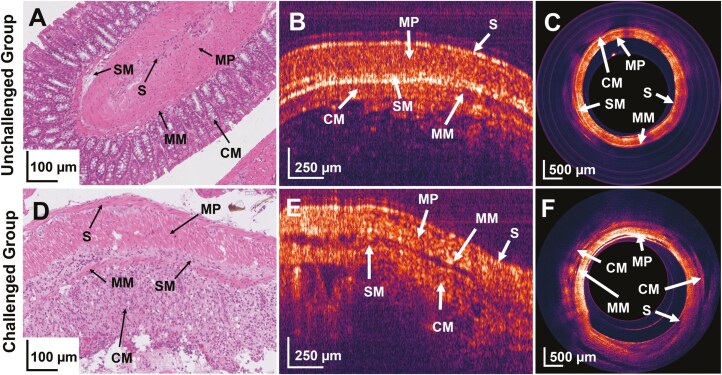
Comparative analysis based on histological and OCT images. **A,** Histological image of control mice colon. **B,** OCT image of control mice colon. **C,** Representative cross-sectional OCT image of unchallenged mice colon. **D,** Histological image of the inflamed colon at day 9. **E,** OCT image of colitis-induced mice colon. **F,** Cross-sectional OCT image of challenged mice colon. CM, colonic mucosa; MM, muscularis mucosa; MP, muscularis propria; OCT, optical coherence tomography; S, serosa; SM, submucosa.

As shown in Figure 5, the microstructures present in mucosa get distorted in inflammatory settings in the challenged group of mice. The OCT images in Figure 5E and F) showed a high degree of inflammation in the colon compared with unchallenged mice. Histological staining of tissue samples from the challenged mice confirms increased gut inflammation (Figure 5D), with barely preserved mucosal architecture, extended ulcerations, increased inflammatory cell infiltrates, and destroyed crypts. Similarly, OCT images revealed that the mucosa and submucosa layers started to separate in challenged mice. The colonoscopic OCT image shown in Figure 5C and F gives the one-shot cross-sectional view of the colon condition. Mark of ulceration, separation of mucosa, and submucosa layers can be seen in Figure 5F.

### Quantitative Analysis Based on OCT Imaging

Furthermore, a quantitative approach was taken using segmented and flattened images to analyze the degree of inflammation. The inflammation led to an increase in the thickness of the colon. Based on quantitative analysis, we further discuss morphological changes due to inflammation.

**Figure 6.**
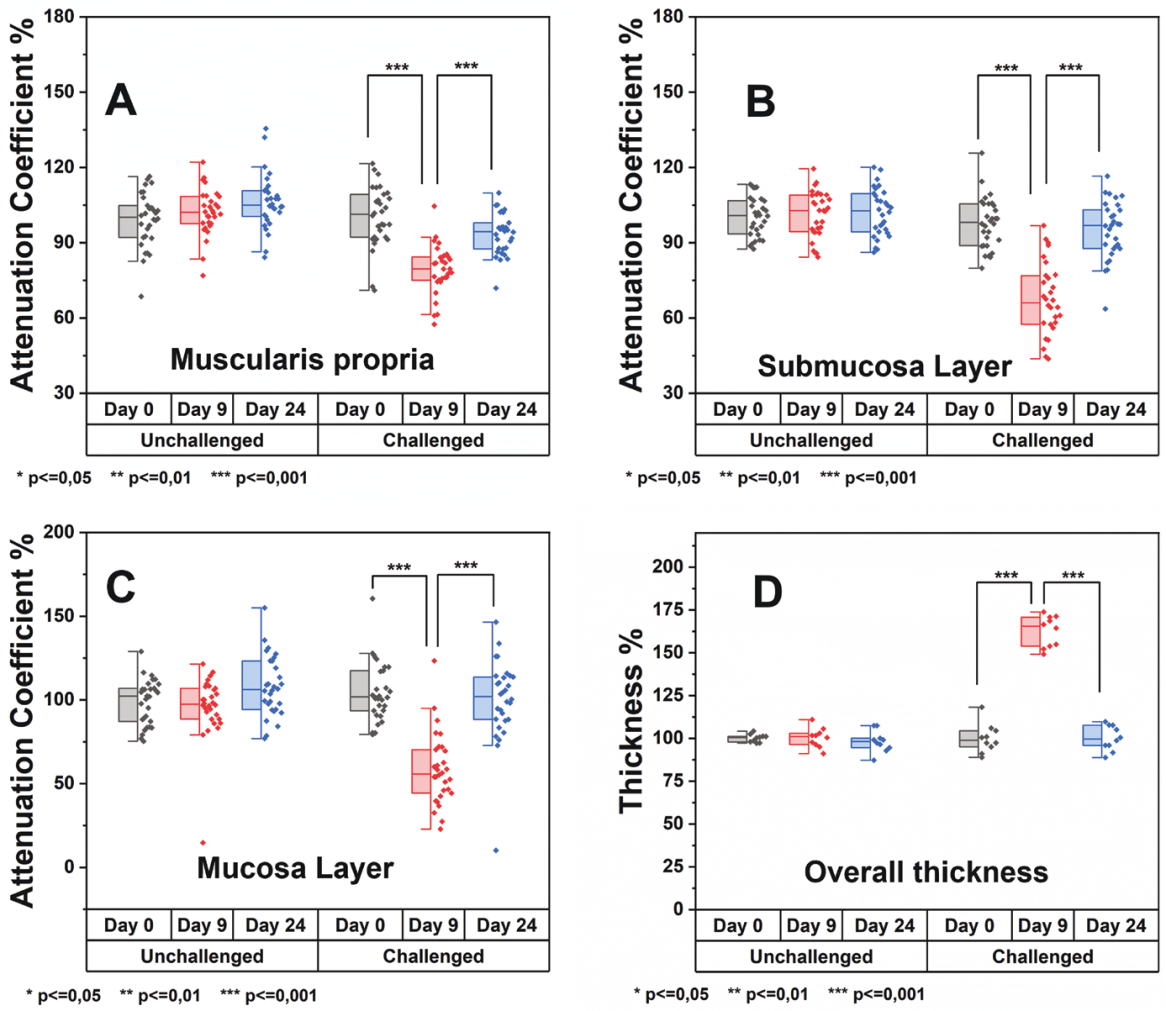
Analytical results showing attenuation coefficient plots of different layers of mice colon and overall thickness change. **A,** The attenuation plot of muscularis propria layer corresponds to the change in percentage with respect to time and experimental subjects. **B,** Attenuation coefficient of submucosal layers. **C,** Attenuation coefficient plot of mucosa including epithelium. **D,** Plot for overall thickness change of colon wall of the mice (*** shows the significant difference).

Inflammation results from the body’s immune response that dispatches white blood cells to the affected site, which is identified as swelling and redness. Optically, a minor change in refractive index can be observed in this case. A quantitative analysis of the attenuation coefficient was performed over different layers of the colon, as shown in Figure 6, along with overall thickness change due to swelling caused by colitis.

Optical coherence tomography imaging allows to visualize 4 different layers of mice colon with different morphological and vasculature features. We quantify the change in attenuation coefficient across colonic mucosa, muscularis mucosa, submucosa, and muscularis propria layers. Figure 6A shows the attenuation coefficient variation for the colonic mucosa for experimental days. The box plots are grouped into control and test mice in the timeline that are day 0 (the initial condition taken as the reference point for normalization of data point), day 9 where the DSS-induced colitis was at its peak, and day 24 was the end of the experiment, showing the recovery of mice from colitis. A significant difference was observed on day 9 of the test mice dataset, showing that attenuation coefficient values decreased significantly and the overall thickness of the colon increased due to swelling. Attenuation coefficient values are calculated at 10 different points along the pullback length of the colon for a single mouse. The graph shows the data for the 3 control and test groups of mice. Similarly, the overall thickness was calculated along the length of the colon and then plotted.

## Discussion

Optical biopsy and IBD diagnosis can be remarkably improved by swept-source OCT colonoscopy; it enables in vivo investigation in real-time with 3D visuals of the colonic wall. Various research groups have used endoscopic OCT imaging for the clinical study of the gastrointestinal (GI) tract18, including the esophagus,^18,19^ stomach,^20^ small and large intestine,^21,22^ and bile duct.^23^ The first in vivo endoscopic OCT was demonstrated by Guillermo J. Tearney in 1997. That showcased the high-speed OCT imaging of pulmonary and GI tracts in the rabbit using a 1 mm diameter fiber optic catheter.^24^ Since then, many OCT endoscopes have been developed and demonstrated.^25^ Side viewing endoscopes with helical scanning have added advantages and gained much popularity among others.

We demonstrated an in-house fabricated endoscopic probe based on a 1310 nm SS-OCT system to assess IBD in mice. The current system can provide 3D images of the colon to identify the potential difference between normal and colitis-induced colon tissue. However, the current system cannot resolve micron-level substructures due to its limited resolution (~10 µm axial and 40 µm lateral). Still, with the help of an analytical approach, we can diagnose pathological changes induced by colitis. Furthermore, we discuss how the current system significantly identifies colitis with a noninvasive, depth-resolved optical biopsy technique compared to white light endoscopes and histological biopsy techniques.

Swept-source optical coherence tomography image acquired through an endoscope probe can showcase the cross-sectional view of the complete colon with colonic mucosa, muscularis mucosa, submucosa, and muscularis propria. An endoscopic view of the OCT image gives a better perspective for visualizing a cylindrical-shaped colon. The impact of IBD causes mucosal erosion, congestion, crypt distortion, micro abscess, dilation, and ulcerations. We observe the deformation of crypts with the OCT enface images shown in Figure 4 in a noninvasive way in in vivo conditions. We quantify the physiological and morphological changes caused by colitis through changes in optical scattering properties and insight into changes in refractive index.

Due to inflammation, the body’s immune response supplies large numbers of white blood cells to fight against the inflammation caused by any means (colitis in this context). The white blood cells have refractive index values of ~1.42, slightly higher than red blood cells and (or) blood (~1.37 to 1.4); the backscattered optical signal registered a slight change in refractive index. Light propagation through scattering media can be understood by the Beer-Lambert law, which defines light attenuation through a media as a combination of scattering and absorptions. These parameters correlate directly with the light wavelength and the media’s refractive index. In the context of the current study, the wavelength of the imaging beam (1310 nm) is in the range of minimal absorption in tissue. So, light’s attenuation coefficient directly depends on the scattering coefficient, which is correlated with the refractive index of scattering media. The graph in Figure 6A-C shows the lowest attenuation coefficient at day 9 for the test group mice with colitis compared to control mice. A blood vasculature network is more prominent in the colon’s muscularis mucosa and submucosa layers than in the colonic mucosa. As biological systems have different attenuation coefficients depending on the type of tissue and fluids,^26^ we believe that the significant decrease in attenuation in the mucosal layer of mice in acute DSS (day 9) comes from the dysregulations in the protective mucus layer and altered concentration of small solutes and macromolecules once the epithelial barrier is disrupted.^27,28^ Restructuring the barrier integrity during the recovery stage increases the attenuation coefficient to a similar value as for the unchallenged mice. Similarly, attenuation in colonic muscle is decreased during inflammation, signaling a loss of strength often observed in IBDs.^29,30^ The colonic mucosa layer has crypt structures that are deformed due to colitis and can be observed by looking closely at the comparison images (Figure 5). Inflammation also causes swelling, and increasing colon thickness, which can be seen through images (Figure 5) and quantitative thickness measurements (Figure 6D).

The results were also verified through gold standard clinical practice for colitis diagnosis: white light endoscopy-based inflammation score results and the physical weight measurement. Both the results are comparable and show that mice suffered from colitis on the day 9 test, and after day 9, the mice recovered slowly. On day 24, test mice recovered from colitis significantly, and all the diagnostic results showed the nearly normal condition of test mice.

In the insight of the above study, we fabricated an endoscopic probe in-house along with a custom-developed RJ. The side viewing probe has a cylindrical diameter of 2.3 mm with an outer protective sheath. Due to its flexibility, smaller thickness, and larger imaging length, the endoscopic probe can image a big portion of the gut in a relatively fast way over commercially available white light endoscope probe. We also developed a segmentation algorithm based on a computer vision image processing tool to remove different artifacts present in the OCT images, like the outer sheath, nonuniformity in the acquired B-scan, and motion artifacts. In the rotating endoscope probe, motion artifacts are very common due to tissue motion between 2 consecutive B-scan images. Due to motion artifacts, the 3D stitching of images gets affected. We use a computer vision tool in MATLAB to develop a segmentation program for segmenting the colon from the OCT B-scan images. After segmentation, we flatten the image and put it on one level. Hence, the primary concern with motion artifacts is minimized.

There are certain limitations to the system that have a scope for improvement. First, the lateral resolution of the system is around 20 µm, which can be improved with GRIN lens-based designs.^31^ Second, the system’s performance is affected by the tissue movement. We tried to reduce such artifacts by image processing, but methods for fixing the tissue during the imaging will significantly improve the image quality. From a technical point of view, the system is ready for human subjects and would require minimal modifications. Since the diameter of the human intestine is much larger than that of a mouse, one modification that we can think of is some probe-centering mechanism. This can be easily achieved with a dilation balloon or a large-diameter clear plastic tube.

## Conclusion

This study demonstrates an in vivo method for diagnosing the progression of IBD in mice using an endoscopic OCT system. The system’s ability to perform OCT diagnostics establishes it as a potential clinical tool for assessing colitis. Disease progression is monitored in real-time and noninvasively with remarkable accuracy. A quantitative approach was employed to estimate the thickness of the tissue and the attenuation coefficient, providing valuable insights into the inflammation caused by colitis. The diagnostic techniques used in this study were compared with established methods for diagnosing colitis, including in vivo white light endoscopy, histological ex-vivo analysis, and physical weight measurements. The comparison indicates that the current endoscopic technique allows for noninvasive, real-time monitoring of disease progression. Additionally, the ability to generate a 3D visualization of the entire colon thickness offers an advantage over traditional white light endoscopy. Improving resolution could yield more detailed information regarding the micro and sub-micron structures within the colonic mucosa.

## Data Availability

The data supporting this study’s findings are available from the corresponding author upon reasonable request.
